# Rates and correlates of psychological distress and PTSD among persons with physical disabilities in Cambodia

**DOI:** 10.1186/s12939-023-01842-5

**Published:** 2023-02-10

**Authors:** Alan Maddock, Paul Best, Nil Ean, Cherie Armour, Nerrolyn Ramstrand

**Affiliations:** 1 School of Social Policy, Social Work and Social Justice, University College Dublin, Dublin 4, Ireland; 2 School of Social Sciences, Education and Social Work, Queen’s University Belfast, Belfast, Northern Ireland; 3 School of Psychology, The Royal University of Phnom Penh, Phnom Penh, Cambodia; 4 School of Psychology, Queen’s University Belfast, Belfast, Northern Ireland; 5 School of Health and Welfare, Jönköping University, Jönköping, Sweden

**Keywords:** Mental Health, Physical Disability, Psychological Distress, PTSD, Cambodia

## Abstract

**Background:**

Compared to the general population, persons with disabilities are at increased risk of poor mental health. The aim of this study was to determine the rates and correlates of psychological distress and post-traumatic stress disorder (PTSD) among persons with physical disabilities in Cambodia.

**Methods:**

From July to December 2021 data were collected as part of a mental health screening programme for persons with physical disabilities who access prosthetic and orthotic services. Psychological distress was measured using the Kessler-10 (K-10) and PTSD using the PC-PTSD-5. Bivariate and multiple linear regression analyses were conducted to identify factors associated with levels of psychological distress and PTSD among this population.

**Results:**

Our study found a high prevalence of psychological distress and PTSD in this patient cohort. Of the 213 participants, 31.5% were likely to be experiencing mild to moderate psychological distress indicative of a mental health disorder, with 13.6% likely to have a severe mental health disorder. Sixty-five percent of patients reported experiencing PTSD symptoms, with forty-six percent meeting the criteria for probable PTSD. Psychological distress was associated with pathological worry, rumination, and facets of mindfulness. Rumination and pathological worry were found to be significant predictors of psychological distress. PTSD symptoms were associated with pathological worry but not facets of mindfulness or rumination. Facets of mindfulness and pathological worry were found to be significant predictors of PTSD.

**Conclusion:**

Integration of mental health services within the disability sector is required to address psychological distress and PTSD symptoms among people with physical disabilities in Cambodia. Health system interventions, such as screening, referral, and the training of health providers, need to be strengthened. Further studies focussing on the psychosocial determinants of mental health of persons with disabilities in Cambodia are required.

## Introduction

Globally, mental health issues and disorders are a significant public health burden [38, 55]. The mental health of a person is influenced by a range of biological, psychological, and social factors, which may be exacerbated by the environment in which people live [56]. Cambodia has a history of genocide and mass violence, with those who experienced the Pol Pot genocide continuing to suffer high rates of psychological distress, trauma, psychiatric morbidity, and poor physical health [37]. Daily stressors in Cambodia such as food scarcity, financial worry, family concerns, and fear of landmine injuries also significantly contribute to the high rate of poor mental health and functional impairment experienced by Cambodian citizens [6]. Other social factors such as child abuse, sexual exploitation, intimate partner violence, trafficking and addictions have also been found to be associated with poor mental health in Cambodia [49]. Persons with mental health issues and disorders in Cambodia are among the most vulnerable and socially excluded citizens [11, 35], who are often overlooked and excluded from social and economic activities, and from exercising fundamental human rights. As a consequence of having mental health issues, many experience stigma and discrimination, all of which can result in lower self-esteem, hope, and motivation [11, 35].

The negative psychological, social, and economic impact of having a mental health issue or disorder is exacerbated if the person also experiences a physical disability [36, 45]. Persons experiencing physical disabilities in developing countries have been identified as having a significantly higher risk of living in multidimensional poverty, which includes both monetary (consumption expenditure) and non-monetary (e.g. living conditions, educational attainment and employment) aspects of poverty, at both household and individual levels [36]. Cambodia, a lower-middle-income country, has very high rates of poverty, with 19% of the population living below the poverty line [46]. Persons with physical disabilities in Cambodia experience the same forms of social exclusion as persons with mental health issues and disorders [45], making it even more likely that they will experience chronic poverty and social isolation [18, 45]. Persons with mental health issues and/or disabilities also face greater barriers to accessing health care than those without disabilities [35, 40]. In the US, adults with disabilities have reported prevalence rates of mental distress which are five times higher than that of persons without disabilities [12]. In lower-to-middle income countries like Cambodia, there is limited knowledge about the patterns of psychological distress and PTSD among persons with disabilities. There are very limited prevalence estimates of the mental health of persons with disabilities in Cambodia. This is despite consistent calls for epidemiological and aetiological research on the mental health of Cambodia citizens [26, 47]. There is only one, small scale, non-peer reviewed study, conducted by Vangkiatkajorn [54] which reported on the rate of comorbid physical disabilities and mental health issues in Cambodia. This study (*n* = 71) investigated rates of depression, anxiety and stress in persons with physical impairments and found that both women (*n* = 27) and men (*n* = 44) experienced extremely severe levels of depression, using the DASS-21. Some gender differences were apparent in that females with physical impairments showed severe levels of anxiety and stress, whereas males reported moderate anxiety and extremely severe stress levels.

Cambodia has very limited mental health service coverage [56] and the increasing evidence of the link between having a disability and a comorbid mental health issue or disorder [12] means that it is likely that persons with disabilities in Cambodia are experiencing high rates of psychological distress and unsupported PTSD. In line with UN Sustainable Development Goal (UN SDG) 3 (ensure healthy lives and promote well-being for all) and Goal 10 (reduce inequality within and among countries) [2], in order to identify if this is the case and to help fill the mental health prevalence data gap, the leveraging of existing disability services in Cambodia to screen the mental health of persons with physical disabilities is particularly important. Also, in line with UN SDG Goal 3 and 10 [2] there is a need to extend mental health services to community-based, primary care settings using task-shifted programmes of mental health support. Such programmes should be informed and evidenced by epidemiological trends and efficacy data [31, 56]. There is however a significant lack of data on what the potential predictors of psychological distress and PTSD symptoms of persons with disabilities in Cambodia might be [26]. Previous research, which have included randomised controlled trial, longitudinal and cross sectional designs, have demonstrated that three psychological processes, rumination [5, 17, 22], worry [23, 25, 29, 39] and mindfulness [19, 43] may be particularly pertinent mechanisms of change of psychological distress and PTSD symptoms, during mental health programmes. No research however has examined if these psychological processes could predict psychological distress and PTSD symptoms of persons with physical disabilities in Cambodia.

In line with UN SDG 3 and 10 [2], the aim of the study was to provide a greater understanding of the rates and correlates of psychological distress and PTSD symptoms of persons with physical disabilities in Cambodia. In order to achieve this aim, this study had three objectives:

1. identify the rates of psychological distress and PTSD symptoms from a sample of data collected from persons with physical disabilities receiving prosthetic and orthotic services in Cambodia;
2. identify individual differences in psychological distress and PTSD symptoms in this sample, and;
3. to examine if there are statistically significant associations between pathological worry, rumination, facets of mindfulness, psychological distress and PTSD symptoms in persons with physical disabilities in Cambodia.

## Methods

### Study setting and design

A community-based cross-sectional survey was conducted from July to December 2021 (six months). Data were collected as part of an innovative mental health screening programme delivered by a local Non-Governmental Organisation (NGO) which supports persons with physical disabilities who require prosthetic or orthotic devices in Cambodia. Patients can attend this NGO’s clinics for a range of reasons e.g., having experienced a recent or historic traumatic injury, due to being born with an impairment, or for review and maintenance of their assistive devices. The screening programme allowed for clinical providers of prosthetic and orthotic devices (clinicians) who work for the NGO to screen the mental health of their patients as an additional component of their standard patient consultation, using the Kessler Psychological Distress Scale [28]. Data on rates of PTSD [42], rumination [53], pathological worry [4] and facets of mindfulness [10] were also collected as part of this process, using validated measurements. The clinician collected these data by asking the patient to answer the questions on each measurement tool, using a wireless tablet, which was connected to an online cloud-based software platform (Qualtrics, Provo, UT). These data were collected at three different NGO sites, Phnom Penh, Kampong Chhnang and Sihanoukville. These sites were selected as they have good internet connectivity. After screening was completed, patients who were deemed to be at risk of mild, moderate or severe mental health problems using the Kessler Psychological Distress Scale [28] were referred to appropriate mental health services.

### Screening and training

The clinicians recruited to act as the screeners/data collectors in this study received two days of training on: 1) ethical principles in research, including consent and confidentiality procedures, 2) mental health issues and disorders, including how to identify signs and symptoms of common mental health issues and disorders (e.g. anxiety and depression), 3) the mental health screening tools that would be used and how to interpret them, and 4) risk assessment, support and referral processes. This training was developed by AM and NE (reviewed by PB and CA), and subsequently delivered in Khmer, the national language of Cambodia, by NE. After the training, the screeners were provided with consistent support by AM and NE. The screeners were encouraged to communicate any issues that might occur during the screening process to AM and NE for resolution. To test the general feasibility and acceptability of the screening, and data collection processes, data were collected from twenty patients (not included in this study). No issues were reported.

### Participants

A power calculation was carried out using G-Power [15] and found that to achieve a power of 0.95 to detect a moderate correlation of 0.3 between the predictor variables and outcomes under investigation (using Pearson *r*), a minimum sample of 200 participants would be required. A purposive and convenience sample of 213 patients (*M* age = 45 years; *SD* = 12.4 years; range = 18–79 years, males = 166; females = 47) was drawn from patients as they awaited their appointment with their prosthetist or orthotist.

### Measurements

#### Psychological distress: Kessler Psychological Distress Scale (K-10)

Psychological distress was measured using the 10-item K10 scale, which measures non-specific distress related to feelings of anxiety and depression [1, 28]. The K-10 was selected due to it being validated in Khmer [7] and its capacity to categorise patients as likely to be well (score < 20), likely to have a mild distress (score = 20–24), likely to have moderate distress (score = 25–29) and likely to have a severe distress (score ≥ 30) [1]. This categorisation allowed the screeners to facilitate a discussion about a subsequent referral to appropriate mental health support services or supports. The reliability of the K-10 was measured using its Cronbach alpha, the most widely used measure of scale reliability [52]. Cronbach alpha values of 0.7 or higher indicate acceptable internal consistency [51]. The K-10’s Cronbach’s alpha in the present study was 0.95.

#### PTSD: primary care PTSD screen for DSM-5 (PC-PTSD-5)

The PC-PTSD-5 was used to screen the patients for PTSD symptoms. The PC-PTSD-5 contains yes/no questions which assess the presence of re-experiencing, avoidance, numbing/attachment, arousal symptoms and trauma-distorted blame and guilt symptoms in the past month [42]. The PC-PTSD-5 has been validated with a US veteran primary care population, with a cut off score of 3 being identified as optimally sensitive for identifying individuals with PTSD in a primary care setting [44]. The PC-PTSD-5’s Cronbach’s alpha for the present study was 0.77.

#### Rumination: Rumination Reflection Questionnaire (RRQ)

Rumination was measured using the 12-item subscale from the Rumination Reflection Questionnaire [53]. This scale measures the extent to which participants are disposed to engage in repetitive thinking about their past (rumination). Higher scores (ranging from 12–60) on RRQ-rumination indicate higher levels of rumination. Trapnell & Campbell [53] reported a high coefficient alpha for this subscale of 0.9, along with good convergent validity with its respective factor predicted from the Big Five factor model of personality (Rumination with Neuroticism; 53]. The RRQ’s Cronbach’s alpha for the present study was 0.86.

#### Pathological worry: the 3-item Penn State Worry Questionnaire (PSWQ-3)

The 3-item Penn State Worry Questionnaire (PSWQ-3) was used to measure pathological worry. The PSWQ-3 has been found to be particularly good measure of pathological worry, and to have comparable internal consistency along with convergent and discriminant validity to the longer 16-item PWSQ, in screening for diagnosis of anxiety disorder [4]. The PSWQ-3’s Cronbach’s alpha for the present study was 0.88.

#### Facets of mindfulness (acceptance and present focus): Chinese version of the Cognitive and Affective Mindfulness Scale — Revised – (CH-CAMS-R)

In order to measure acceptance and present focus, two key facets of mindfulness which are relevant to psychological distress and PTSD, the acceptance and present focus subscales (both being two item scales) of the Chinese version of the Cognitive and Affective Mindfulness Scale were used. Each item was measured by a 4-point Likert scale, with a minimum of 2–8 for each subscale, with higher scores relating to higher levels of acceptance and present focus. The two subscales were also combined to give an overall facets of mindfulness score, ranging from 4 to 16. The Ch-CAMS-R has been found to obtain good levels of reliability, validity and factor structure as the original CAMS-R [10]. It was also found to have good convergent validity with the DASS-21 [10]. The Cronbach’s alpha for the 4-item facets of mindfulness scale for the present study was 0.85.

For the purposes of this study the PC-PTSD-5, the RRQ, the PSWQ-3 and CH-CAMS-R were translated into Khmer by NE.

### Data analyses

Analyses were performed using SPSS 27.0 (IBM, Armonk, NY). The data were screened for missing values and for potential outliers. Potential outliers were measured using the interquartile rule [24], using whisker and plots on SPSS 27 (IBM, Armonk, NY). No outliers were found, and there was no missing data. Two separate correlation and multiple linear regression analyses of each of the potential predictor variables (pathological worry, rumination, facets of mindfulness) of psychological distress and PTSD were conducted. All regression assumptions were examined for inferences to be valid. Scatterplot diagrams were inspected, and linear relationships were found between each of the predictor variables and outcomes. Assumption of independence of residuals was assessed using the Durbin-Watson statistic [13]. The Durbin-Watson statistic ranges from zero to four, with a value of approximately two indicating no correlation between residuals. There was independence of residuals, as demonstrated by a Durbin-Watson statistic of 1.7 for the regression on psychological distress and 1.5 for the regression on PTSD. To assess Homoscedasticity, the plot of standardised residuals versus the standardised predicted values was visually inspected. These residuals were normally distributed as indicated by a normal probability plot. There was no multicollinearity, all tolerance values were > 0.1 and all variance inflation factor values were less than 10 [21]. Visual inspection of the Q-Q Plots found that the assumption of normality of residuals was met.

### Ethical statement

As part of their clinical consultation, screeners advised patients of the study’s objectives and ascertained their interest in taking part. If the patient was interested, written consent was attained after the patient was made aware that the study was voluntary in nature and that they could discontinue their participation at any point. The screening tools were then administered. This study was approved by the National Ethics Committee for Health Research of the Ministry of Health, Cambodia approved (Reference no. 033 NECHR) and Queen’s University, Belfast Research Ethics Committee (REF 028_2021).

## Results

### Psychological distress

In total, 31.5% (*n* = 67) of the patients had mild to moderate levels of psychological distress, with 13.6% (*n* = 29) experiencing severe psychological distress. Results from the Pearson correlation tests indicated a significant positive association between facets of mindfulness (*r* (213) = 0.29, < 0.001), rumination (*r* (213) = 0.32, < 0.001), worry (*r* (213) = 0.54, < 0.001) and psychological distress. Multiple linear regression analysis was used to test if facets of mindfulness, rumination, pathological worry significantly predicted psychological distress. A significant regression was found (F (3, 209) = 32.69, *p* < 0.001, *R*2 = 0.32, 95% CI [0.21, 0.40]). The individual predictors were further examined and indicated that rumination (*b* = 0.191, t = 3.2, *p* = 0.002) and pathological worry (*b* = 0.51, t = 7.25, *p* =  < 0.001) were significant predictors in the model. Facets of mindfulness did not significantly predict psychological distress (*b* = -0.15, t = -0.8, *p* = 0.45).

### PTSD

Sixty-five percent of participants (*n* = 138) reported experiencing symptoms of PTSD, with forty-six percent of participants (*n* = 97) likely experiencing PTSD (with a score of 3 or more on the PC-PTSD-5). Results of the Pearson correlation tests indicated a significant positive association between pathological worry (*r*(136) = 0.4, *p* < 0.001) and PTSD, but not facets of mindfulness (*r*(136) = 0.03, *p* = 0.74) or rumination (*r*(136) = 0.13, *p* = 0.15). Multiple linear regression analysis was used to test if facets of mindfulness, rumination, pathological worry significantly predicted PTSD. A significant regression was found (*F*(3, 132) = 10.3, *p* < 0.001, *R*2 = 0.19, 95% CI [0.07, 0.28]). The individual predictors were further examined and indicated that facets of mindfulness (*b* = -0.195*,* t = -2.2, *p* = 0.03) and pathological worry (*b* = 0.47, t = 5.3, *p* =  < 0.001) were significant predictors in the model. Rumination did not significantly predict PTSD (*b* = 0.05, t = 0.6, *p* = 0.57).

## Discussion

The aims of this paper were to identify the rate of psychological distress and PTSD among a purposive and convenience sample of persons with physical disabilities, and some of the variables which may predict both outcomes. Our main results show that 31.5% of the participants screened were experiencing mild or moderate psychological distress, with 13.6% meeting the criteria for severe psychological distress. Sixty-five percent of the study sample experienced PTSD symptoms, with forty-six percent of the participants also meeting the criteria for probable PTSD. This study also found that levels of pathological worry and rumination predicted levels of psychological distress in this group of persons with disabilities who are orthotic or prosthetic service users. PTSD symptoms were found to be predicted by pathological worry and mindfulness but not rumination levels in this sample.

The overall rates of psychological distress (45.1%) in this sample of persons with disabilities was higher than Seponski et al. [48] found in a large representative sample of Cambodian adults. Seponski et al. [48] found very high rates of anxiety (27.4%) and depression (16.7%) in the general Cambodian population, which are much higher than the global population prevalence of anxiety (approximately 7–8%) and depression (approximately 10%) [3, 9]. We also found much higher rates of probable PTSD (46%) than Seponski et al. [48] (7.8%). Some of the variation in our results may be due to the different measurements, sampling, and recruitment methods used in our study and Seponski et al. [48]. However, the variation is more likely due to comorbid physical disabilities in our study population. This is supported by Cree et al. [12] who, in a US context, found that 32.9% of persons with disabilities in the USA reported experiencing mental distress, with 38.6% of those who were unemployed, and thus perhaps living in poverty, experiencing mental distress. Due to the data collection restrictions in this study, we did not collect data on the income levels of our sample, however it is likely that most of the sample were living in poverty. The fact that our sample were likely experiencing poverty along with a physical impairment, and are living in a post-conflict nation, with a recent tragic history, where very limited mental health supports exist, or are inaccessible [41], help to explain the even higher rates of psychological distress and PTSD in our sample versus those found in Cree et al. [12]. In line with the World Health Organization [9] and the United Nations (UN) 2030 Agenda for sustainable development [2], particularly goals 3 and 10, mental health policies and service development strategies need to focus on the most vulnerable groups. This study highlights how persons with disabilities in Cambodia are a particularly vulnerable group who likely experience a very high prevalence of mental health issues and disorders. The mental health policies and development strategies should thus include provisions and key mental health action plans focussing on persons with disabilities, e.g., increasing social inclusion activities, such as increasing access to education and employment opportunities [48].

Persons with disabilities often experience additional barriers to accessing health care [35, 40]. This issue is exacerbated in LMICs, such as Cambodia, where there are chronic shortages of trained professionals and limited access to mental health services [26]. The results from this study highlight, in line with Eaton et al. [14] and Yi et al. [56], that training on common mental health issues and disorders, and the task shifting of mental health screening to other professionals, such as prosthetists or orthotists, is a potentially effective way to ensure that the unmet mental health needs of persons with disabilities are identified. The screening of the mental health of persons with disabilities in this study allowed clinicians to identify mild to moderate mental health issues earlier and refer to preventative services and supports, while referring patients with more severe symptoms or disorders to specialist mental health services in Cambodia [56]. The decentralisation of mental health services in Cambodia, which are typically heavily concentrated in urban areas, has consistently advocated for [26, 47, 49, 56]. The expansion of the mental health and screening programme to other organisations supporting persons with disabilities in Cambodia, could help facilitate the decentralisation of mental services to rural communities in Cambodia. For organisations supporting persons with disabilities to integrate such mental health screening programmes as part of routine practice, additional health funding would be required by international donor agencies or by the Cambodian government.

In line with international literature, pathological worry was found to be significant predictor of psychological distress in this sample of persons of physical disabilities. This finding is supported by Holditch-Davis et al. [25], Nikcevic et al. [39] and Liu et al. [29] who, using cross-sectional and longitudinal methodologies, found that worry predicted the psychological distress of a range of health, mental health and non-clinical populations across a range of age ranges. The rumination levels of this sample of persons with disabilities was also found to be a significant predictor of psychological distress. This finding is supported by Galfin and Watkins [17], Buelens et al. [5] and Hill and Watkins [22], who using cross-sectional and longitudinal methodologies, also found that rumination was a significant predictor of psychological distress in patients in palliative care, self-harming adolescents, and patients with ovarian cancer, respectively. Both worry and rumination have been identified as cognitive avoidant coping strategies, which may relieve stress, anxiety, or low mood in the short term, but if relied upon in the longer term, will likely increase psychological distress, as the underlying reasons for the worry or rumination e.g., not approaching and engaging with difficult emotions, are unlikely to change [30, 32, 33]. These results provide promising preliminary evidence that should a person with disabilities in Cambodia engage in a mental health intervention or programme of support, which could reduce worry and rumination e.g., mindfulness-based programmes, Cognitive Behavioural Therapy or task shifted health worker counselling support programmes [31], that they are likely to experience improvements in psychological distress.

Pathological worry was found to be a significant predictor of PTSD in this sample of persons with disabilities. This helps to validate Hinton and Bui [23], who outline the importance of worry as a potentially key variable in the development of PTSD symptoms in the Cambodian context. Our results are also supported by Liu et al. [29] who found that worry in a young adult mental health population was significantly associated with clinical levels of PTSD symptoms. Our study also found that two key facets of mindfulness combined (having a present focus and acceptance of difficulty and things one cannot change) predicted PTSD symptoms in this sample. This is supported by Stephenson et al. [50] who in a secondary analysis of 4 RCTs of Mindfulness-based stress reduction with US army veterans found that increases in mindfulness were significantly associated with reduced PTSD symptoms. Our findings were also supported by Gibert et al. [19] who in a 4.5 year prospective cohort study of terrorist attack survivors, found that levels of mindfulness were significantly associated with PTSD symptoms. These results, again provide promising preliminary evidence that should a person with a physical impairment in Cambodia, engage in a mental health intervention or programme of support e.g., eye movement desensitization and reprocessing (EMDR) and/or mindfulness-based programmes [31], which could reduce pathological worry and facets of mindfulness, that they may experience improvements in their PTSD symptoms.

## Conclusions

This study found very high rates of psychological distress and PTSD symptoms among persons with physical disabilities in Cambodia. Our results suggest that persons with physical disabilities in Cambodia are likely to be at greater risk of developing mental health issues or disorders than those in the general population. We also found that facets of mindfulness, pathological worry and rumination could be relevant mental health variables that could be targeted by mental health interventions in the Cambodian context, in order to improve these very important mental health outcomes. Even though these findings have clinical relevance, they are associated with a number of limitations. As this study stemmed from a pilot mental health screening programme, which was built into the clinical care received by patients, we were limited in the number of questionnaires we could ask the prosthetist/orthotists to complete with the patients. This meant that we had to refine the battery of questionnaires significantly e.g., instead of using the Penn State Worry Questionnaire (which is a 16-item measure) to measure worry, we used the 3-item PSWQ-3 [4] to measure pathological worry, and instead of using the Southampton Mindfulness Questionnaire [8] (which is also a 16 item measure) to measure mindfulness, who used the 4-item CH-CAMS-R, to measure facets of mindfulness. The PSWQ-3 and CH-CAMS-R both proved to be reliable measures of these constructs; however, their more restricted range might have reduced correlations between these predictor variables and the psychological distress and PTSD symptoms of this sample of persons with disabilities [16]. The limited scope of this study also reduced our capacity to examine a range of other potentially important predicting variables of psychological distress and PTSD in persons with disabilities in Cambodia. This more limited scope also meant that we could not examine what the social determinants of the mental health of persons with disabilities in Cambodia could be, nor determine how the type and extent of disability may have influenced mental health outcomes. Future research should examine both the psychological and social determinants of mental health in this population. This would also allow potential social predictors of psychological distress and PTSD symptoms e.g., poverty levels, to be examined. The use of purposive and convenience sampling also limits the generalisability of these findings to a wider population of persons with disabilities in Cambodia [20]. The use of one data collection point also means that conclusions with regard to causality between pathological worry, rumination, facets of mindfulness and psychological distress and PTSD cannot be asserted [27, 34]. In order to overcome this limitation, future research on the mental health of persons with physical disabilities in Cambodia should use longitudinal designs in order to examine changes in psychological distress and PTSD over time. Despite these limitations, this study highlights the feasibility of integrating mental health system interventions, such as screening and referral processes, as part of the disability sector in Cambodia. These task-shifted mental health interventions, facilitated through short clinician training programmes, allowed data on the rates of the psychological distress and PTSD of persons with disabilities to be attained, and for these patients to referred to relevant mental health supports based on their mental health needs. Replication of this process in other parts of Cambodia, could support the decentralisation of mental health services across Cambodia, at a community based, primary care level. This would support the earlier identification of mental health issues and disorders and for preventative supports to be put in place earlier.

## Data Availability

The supporting data can be made available upon reasonable request.
